# The Pacific Medical and Surgical Journal

**Published:** 1858-03

**Authors:** 


### The Pacific Medical and Surgical Journal.

We have received the first number of a Medical Journal with the above title, published in San Francisco, California, and edited by John B. Trask, M. D., and David Wooster, M. D.

"The California State Medical Journal" having been allowed to die out for want of patronage, it would seem somewhat hazardous to embark in another enterprise of a similar character, without some reliable assurance of support. We hope, however, with the adventurous gentlemen who have undertaken it, that the medical men of California "have regretted their negligence in the past, and to save the present Journal from a similar fate, will support it with more promptitude and liberality." A Medical Journal in that distant and somewhat isolated region, we should consider *a necessity*, and judging from the first number, we should say unhesitatingly that this Journal will richly deserve the fullest success. We hope the publishers will be able to number among their subscribers many from this quarter of the United States, to whom it would doubtless be found interesting and profitable, the Editors proposing to make it not only a reliable record of the state of Medicine in California, but to make it a medium for the report of the Practice of the Occidental South American Capitals, of the Islands and Oregon. We welcome the Journal to our exchange list, and hope to be able to chronicle its prosperity. It is published monthly at five dollars per annum, or three dollars for six months, in advance.

